# Neuromuscular Phenotype in Long COVID Is Associated with Inflammation, Mineral Homeostasis, and Muscle Metabolism

**DOI:** 10.3390/v18101099

**Published:** 2026-10-04

**Authors:** Camilla Costa Silva, Karina Carvalho Marques, Pablo Fabiano Moura das Neves, Daniel Carvalho de Menezes, Stanley Soares Xavier, Cristiane de Fátima Pimenta da Costa, Marcio Clementino de Souza dos Santos, Ápio Ricardo Nazareth Dias, Mário Augusto Costa Prazeres, Pedro Fernando da Costa Vasconcelos, Juarez Antônio Simões Quaresma, João Sérgio de Sousa Oliveira, Luiz Fábio Magno Falcão

**Affiliations:** 1 Laboratory of Research and Assistance in Sequelar Infectious Diseases in the Amazon, LabDis, Pará State University, No. 2623, Perebebuí Street, Belém 66087-662, PA, Brazil; 2 Postgraduate Programme in Parasitic Biology in the Amazon, University of Pará State, Belém 66087-662, PA, Brazil; 3 Centre for Biological and Health Sciences, University of Pará State, Belém 66087-662, PA, Brazil; 4 Postgraduate Programme in Rehabilitation and Functional Performance, University of Pará State, Belém 66087-662, PA, Brazil; 5 Department of Arbovirology and Hemorrhagic Fevers, Evandro Chagas Institute, Ananindeua 67030-000, PA, Brazil; 6 Department of Pathology, Paulista School of Medicine, Federal University of São Paulo, São Paulo 04023-062, SP, Brazil; 7 School of Medicine, São Paulo University, São Paulo 01246-903, SP, Brazil

**Keywords:** long COVID, neuromuscular dysfunction, muscle impairment, surface electromyography, ferritin, lactate dehydrogenase, body composition

## Abstract

**Background:** Neuromuscular dysfunction in long COVID remains poorly understood, and conventional biomarkers of muscle injury often fail to account for persistent symptoms. This study aimed to compare laboratory biomarkers and neuromuscular outcomes by sex, long COVID duration, and hospitalisation status, and examine the association between neuromuscular outcomes and laboratory biomarkers. **Methods:** In this cross-sectional study, 153 adults with long COVID underwent clinical, body composition, handgrip strength, surface electromyography, and laboratory assessments, including creatine kinase (CK), lactate dehydrogenase (LDH), ferritin, erythrocyte sedimentation rate (ESR), haemoglobin, sodium, potassium, and calcium. Analyses were stratified by sex, disease duration, and hospitalisation. **Results:** Significant between-group differences in laboratory and neuromuscular variables were more frequent among men than among women. Hospitalised women had lower muscle mass and higher body fat than non-hospitalised women. Men with longer long COVID duration differed in CK, ferritin, haemoglobin, and vastus lateralis electromyographic activity. Ferritin, calcium, potassium, haemoglobin, and LDH showed consistent, modest multivariate associations with neuromuscular outcomes. The haemoglobin–ferritin–ESR axis correlated most consistently with muscle mass and handgrip strength. **Conclusions:** These exploratory findings suggest a sex-specific pattern and a consistent haemoglobin–ferritin–ESR axis in long COVID, requiring confirmation in longitudinal studies.

## 1. Background

Persistent muscle-related symptoms, including fatigue, weakness, myalgia, and reduced exercise tolerance, are among the most disabling manifestations of long COVID and may substantially impair quality of life and physical function [1]. These symptoms, however, are non-specific and may result from systemic alterations associated with long COVID and/or from physical deconditioning secondary to reduced activity, and should not be interpreted, in isolation, as evidence of intrinsic muscle damage. Objective neuromuscular assessments are therefore needed to more precisely characterise the structural and functional components of these manifestations. Although growing evidence supports the presence of neuromuscular involvement in long COVID, the mechanisms underlying these persistent impairments remain incompletely understood [2]. Many patients exhibit persistent muscle dysfunction despite the absence of clear clinical or laboratory evidence of ongoing muscle injury, suggesting that long COVID represents a distinct neuromuscular phenotype that extends beyond isolated structural myopathy. Current evidence suggests that this dysfunction may arise from multiple, potentially overlapping mechanisms, both structural and functional, including critical illness-associated myopathy, altered motor unit recruitment or discharge patterns, mitochondrial dysfunction, impaired oxidative metabolism, and compensatory muscle recruitment strategies secondary to prolonged deconditioning [3,4,5].

Several laboratory biomarkers may provide insights into the biological pathways contributing to post-COVID neuromuscular dysfunction. Ferritin has emerged as a particularly relevant marker because it lies at the intersection of immune activation, iron metabolism, oxidative stress, and mitochondrial bioenergetics, all of which have been implicated in the pathophysiology of long COVID [6,7]. Likewise, disturbances in mineral homeostasis, particularly involving calcium and other electrolytes, may contribute to persistent muscle dysfunction through alterations in excitation–contraction coupling, intracellular signaling, and neuromuscular transmission [8]. Hemoglobin may also be relevant, as impaired oxygen transport can adversely affect muscle oxidative capacity and physical performance [9].

In contrast, conventional biomarkers of muscle injury, such as creatine kinase, have yielded inconsistent findings in long COVID [10]. Although elevations in these markers have been reported during acute SARS-CoV-2 infection and in patients with COVID-19-related myopathy [11], their ability to reflect persistent neuromuscular manifestations after the acute phase remains uncertain. This discrepancy raises the possibility that ongoing muscle dysfunction may be driven more by metabolic, inflammatory, and neuromuscular mechanisms.

Previous investigations of peripheral nerve and muscle function in long COVID have reported limited evidence of overt neuropathic involvement, whereas myopathic and functional alterations appear to be relatively common and may contribute to reduced muscle mass, diminished strength, fatigue, myalgia, and impaired physical performance [4]. However, the interactions between laboratory biomarkers, body composition, muscle function, and neuromuscular activation remain poorly characterized. Furthermore, the potential influence of sex, disease duration, and hospitalization history on these relationships has received little attention.

Therefore, this study aimed to compare laboratory and neuromuscular variables, including body composition, muscle strength, and surface electromyographic activity, between subgroups defined by long COVID duration and hospitalisation status, stratified by sex, and to examine the multivariate association between laboratory biomarkers and these neuromuscular outcomes.

## 2. Materials and Methods

### 2.1. Participants and Consent

The study population comprised adults of both sexes aged ≥18 years who presented with symptoms consistent with acute COVID-19, including fatigue, dyspnoea, myalgia, arthralgia, and other related symptoms. Eligibility criteria required either a positive reverse transcription polymerase chain reaction (RT-PCR) test or a positive antibody test. Participants were evaluated at least 30 days after the onset of initial COVID-19 symptoms, between September 2020 and December 2021, following voluntary enrolment in a clinical follow-up programme for patients with long COVID in the Brazilian Amazon.

The clinical definition of long COVID proposed by Raveendran (2021) [12] was adopted for this study. Although four diagnostic categories were originally described (confirmed, probable, possible, and doubtful), only participants classified as having confirmed or probable long COVID were included in the present analysis. Classification was based on the following criteria: (1) a documented history of SARS-CoV-2 infection; (2) persistent or newly emergent symptoms not attributable to alternative clinical conditions; and (3) a positive result on RT-PCR testing or serological antibody testing.

Study participants were assessed by a multidisciplinary medical team through clinical interviews and the completion of a specific protocol form designed to gather information on pre-existing conditions (cardiovascular, pulmonary, infectious, and metabolic diseases), data on the current illness (symptomatology, medication use, sequelae, etc.), and socio-economic data (educational level, marital status, income, etc.). This comprehensive clinical evaluation, together with the diagnostic tests performed and the medical history collected, enabled the exclusion of alternative diagnoses that could account for the reported symptoms, thereby supporting the classification of participants as presenting long COVID.

Patients with a documented history of neurological conditions (e.g., stroke, among others), musculoskeletal conditions (such as osteoarthritis, disc herniation, fibromyalgia, spinal surgery, ligament rupture, fractures, tendinopathy, etc.), cardiac conditions (acute myocardial infarction, arrhythmia, etc.), or infectious diseases (such as Human Immunodeficiency Virus [HIV] and Human T-Lymphotropic Virus [HTLV], etc.) were excluded from the analysis. Participants were stratified by sex into study groups based on the duration of long COVID (G ≤ 6: patients with long COVID duration of up to 6 months; G > 6: patients with long COVID duration exceeding 6 months), and hospitalisation during the acute phase (GH: patients who were hospitalised; GNH: patients who were not hospitalised). Participants were classified as sedentary if they reported no regular physical activity and did not meet the World Health Organization (2020) recommendations of 150–300 min of moderate-intensity physical activity, 75–150 min of vigorous-intensity physical activity, or an equivalent combination per week [13].

This study was approved by the Research Ethics Committee of the University of Pará State (Opinion no. 252,664/2020), ensuring compliance with ethical guidelines for human subject research.

### 2.2. Assessment of Muscle Biomarkers

The tests conducted involved the analysis of creatine kinase (CK), lactate dehydrogenase (LDH), ferritin, erythrocyte sedimentation rate (ESR), haemoglobin, calcium, sodium, and potassium levels. After a minimum of 8 h of fasting, 3 mL of blood was collected in two tubes with a separating gel and clot activator for serum analysis. The CMD 600 equipment (Wiener Laboratorios SAIC©, São Paulo, Brazil) was employed, and the quantification of the values for each test resulted from the interaction of the reagents present in the equipment designed for the aforementioned tests (Reagent kits dedicated to clinical chemistry in automatic analyzers—Wiener Lab©), utilizing photometry for quantification. The reference values provided by the reagent manufacturer were used as the reference standard.

### 2.3. Manual Dynamometry

Hand grip strength (HGS) was measured using a hydraulic dynamometer, model Crown (Filizola^®^, São Paulo, Brazil), and was performed in accordance with the recommendations of the American Society of Hand Therapists [14]. The patient was seated with hips and knees positioned at approximately 90 degrees of flexion and feet flat on the floor. The tested limb was positioned with the shoulder in adduction and neutral rotation, the elbow flexed at 90 degrees, the forearm in a neutral position, and the wrist extended between 0° and 30°. Using standardized verbal commands, participants performed a maximal contraction lasting 3 s for each test. Three trials were conducted with 30-s intervals between them. The rest interval between tests for each hand was 2 min. Reference values were obtained using the predictive equation for the Brazilian population proposed by Chagas [15].

### 2.4. Bioimpedance

The percentage of muscle mass (MM) was assessed using a digital scale with bioimpedance HBF–514C (Omron Healthcare Brasil^®^, São Paulo, Brazil). The patients stood on the scale barefoot, distributing their weight evenly and maintaining an upright posture, looked straight ahead, and remained still until the measurement was completed. The reference values were based on the sex and age range criteria recommended by the manufacturer (Supplementary Table S1).

### 2.5. Kinesiological Electromyography

Muscle electrical activity of the anterior deltoid (AD), vastus medialis (VM), and vastus lateralis (VL) was collected using the Miotool 400 electromyograph (Miotec^®^ Biomedical Equipment S.A., Porto Alegre, Brazil). The equipment settings were adjusted to a final gain of 1000 times, with filters that created a passband ranging from 10 to 500 Hz, and a notch filter adjusted to 60 Hz [16].

Signals were sampled at 2000 samples/second per channel using differential surface sensors (SDS500, Miotec^®^) with adhesive conductive hydrogel surface electrodes (44 × 32 mm; Solidor, model MSGST-06, Health Supply©, Osasco, Brazil, Ag/AgCl, rectangular, 10 mm diameter), positioned in pairs with a 25 mm inter-electrode distance. Skin preparation, patient positioning, and electrode placement followed the SENIAM (Surface EMG for Non-Invasive Assessment of Muscles) guidelines.

Patients performed isotonic contractions involving shoulder abduction and knee extension at a constant speed with a data capture window of 30 s. The outcome measure was the standard deviation of the root mean square (RMS), subsequently normalised using the dynamic peak of the signal, and was expressed as a percentage. A higher percentage of electrical activity in the muscle indicated greater recruitment of motor units. This condition may reflect reduced biomechanical efficiency when performing a given task [17]. Given that surface EMG signal quality can be compromised by increased subcutaneous adipose tissue [18], overweight and obese participants (BMI > 25 kg/m^2^) were excluded from the electromyographic analysis.

### 2.6. Statistical Analysis

Data were analysed in R (version 4.5.2; R Foundation for Statistical Computing, Vienna, Austria). Categorical variables were analysed using Fisher’s exact test or the chi-square test, as appropriate. The distribution of continuous variables was assessed using the Shapiro–Wilk test. Between-group comparisons were performed using Student’s *t*-test or the Mann–Whitney U test, as appropriate. Pearson’s or Spearman’s correlation coefficients were calculated according to the distribution of the data.

The multivariate neuromuscular outcome was defined as a six-variable response matrix. It included muscle mass percentage, handgrip strength, and normalised RMS values for the right and left AD and VL muscles. These measures were selected a priori to capture complementary domains of neuromuscular status: body composition, muscle function, and electromyographic muscle activation.

These outcomes were standardised to a mean of 0 and a standard deviation of 1 within each imputed dataset. Euclidean distances were subsequently calculated from the standardised response matrix. The laboratory biomarkers included as predictors were CK, haemoglobin, ESR, ferritin, calcium, sodium, potassium, and LDH. Missing values for these variables were handled using multiple imputation by chained equations with predictive mean matching, generating five imputed datasets. A PERMANOVA model was fitted separately for each imputed dataset using the ‘adonis2’ function from the vegan package, based on Euclidean distances and 4999 permutations. All laboratory biomarkers were entered simultaneously into a single model. Individual biomarker effects were assessed marginally using the ‘by = “margin”’ argument; thus, each pseudo-*F*, partial R^2^, and permutation-based *p*-value was estimated conditionally on the other laboratory biomarkers in the model, rather than sequentially according to the order of entry.

Demographic and clinical covariates—including sex, age, BMI, acute COVID-19 severity, hospitalisation status, and long COVID duration—were not included in the primary PERMANOVA model. Median and range values for partial R^2^ and pseudo-*F* were used as descriptive summaries of result stability across imputations, while complete results for each imputed dataset are presented in Supplementary Table S3. For each biomarker, the number of imputations yielding *p* < 0.05 was also reported.

Artificial intelligence tools were used to support the preparation of this manuscript. The OpenEvidence platform (Miami, USA) was utilised during the literature review process to assist in identifying and summarising relevant scientific evidence. ChatGPT (OpenAI, San Francisco, USA) was subsequently used solely for language editing and editorial support, including improvements in text clarity, coherence, and readability. All study design, data analysis, interpretation of findings, selection of references, and formulation of conclusions were performed independently by the authors. The authors take full responsibility for the accuracy, originality, and integrity of the content presented.

## 3. Results

**Figure 1:**
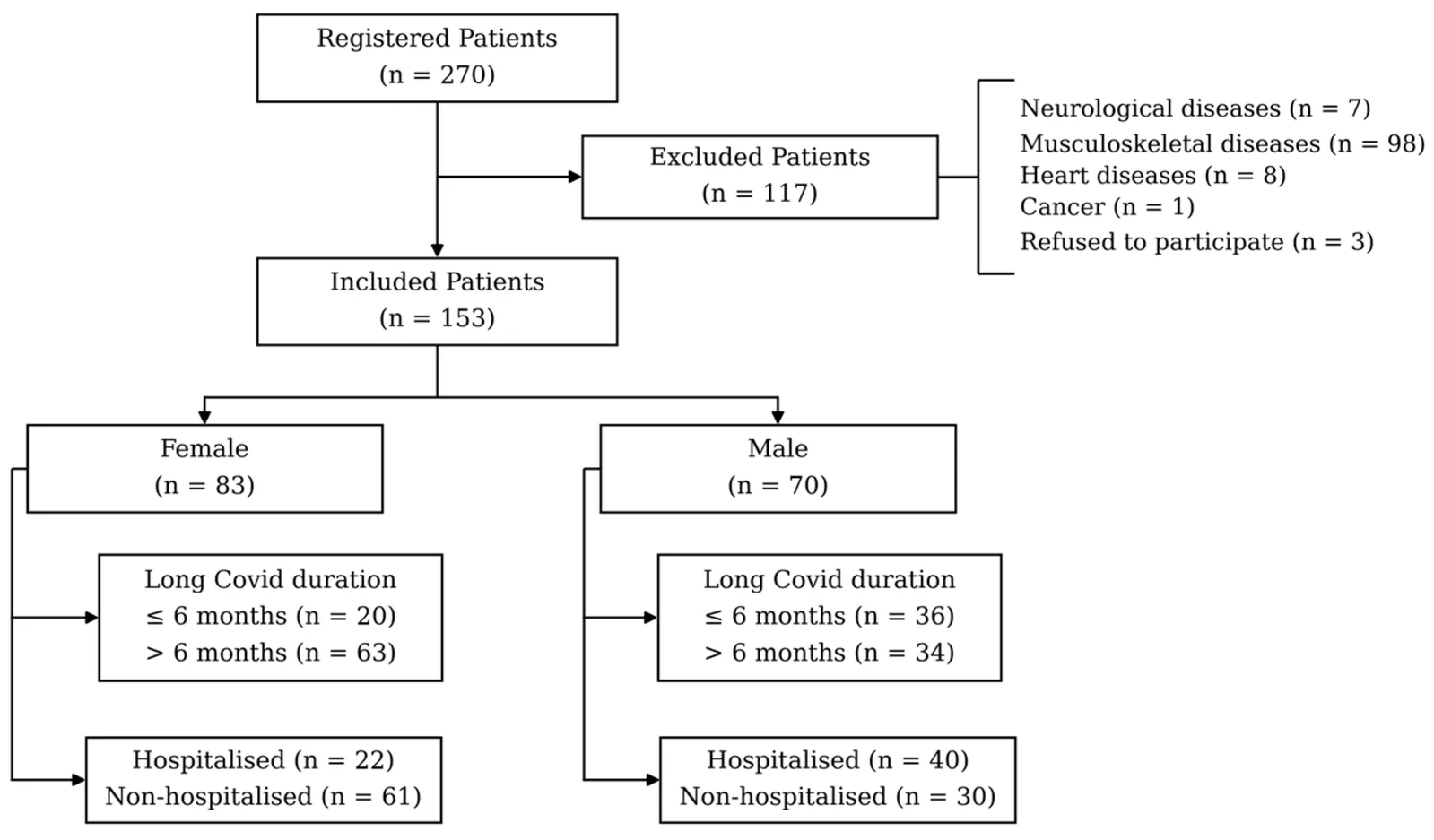
Flowchart of selection and recruitment of patients. Of the 270 registered patients, 117 were excluded owing to predefined eligibility criteria. A total of 153 patients were included in the study. Participants were stratified by sex. They were then assigned to subgroups according to: (1) long COVID duration—G ≤ 6, patients with long COVID duration of up to 6 months; and G > 6, patients with long COVID duration exceeding 6 months; and (2) hospitalisation during the acute phase (GH, patients who were hospitalised; and GNH, patients who were not hospitalised).

Of the 270 patients enrolled in the comprehensive long COVID care programme, 117 were excluded according to predefined eligibility criteria: neurological disease, musculoskeletal disease, cardiac disease, cancer, or refusal to participate. The final study sample comprised 153 patients. Participants were first stratified by sex into women and men. Within each sex stratum, participants were further categorised according to long COVID duration and hospitalisation status during the acute phase of COVID-19 (Figure 1).

The study sample consisted predominantly of female participants, with a mean age of 48 years. Obesity was the most prevalent comorbidity. Hypertension was significantly more prevalent among men than women. Men also experienced more severe COVID-19 cases than women, as shown by higher rates of hospitalisation, longer hospital stays, and increased admissions to intensive care units and receipt of invasive mechanical ventilation. The most reported symptoms were fatigue, dyspnoea, muscle weakness, and myalgia. Several symptoms were reported more frequently by women, including dyspnoea, myalgia, headache, chest pain, and lower-limb oedema (Table 1).

Table 2 presents the comparisons performed between groups stratified by sex and subdivided according to long COVID duration and hospitalisation status, encompassing laboratory and clinical/neuromuscular variables. The corresponding descriptive statistics (median [IQR] or mean ± standard deviation) for each subgroup are presented in Supplementary Table S2.

Among women, ESR differed significantly according to the duration of long COVID, being higher in patients with a disease duration of ≤6 months than in those with a longer duration (55.5 [30.8–66.8] vs. 42.0 [25.8–49.3] mm/h; effect size = −0.286; *p* = 0.044). Regarding hospitalization status, previously hospitalized women exhibited lower muscle mass (22.3% [21.1–23.5] vs. 23.9% [22.4–25.6]; effect size = −0.393; *p* = 0.008) and higher body fat mass (48.0% [44.0–50.7] vs. 40.5% [35.8–47.8]; effect size = 0.380; *p* = 0.010) compared to non-hospitalized women. No other laboratory or clinical variables showed a significant difference between the female subgroups analysed.

Among men, several laboratory and neuromuscular variables differed significantly according to the duration of long COVID. CK and haemoglobin levels were higher in patients with a duration >6 months than in those with ≤6 months (CK: 100 [79–146] vs. 69 [51.5–124] U/L; effect size = 0.299; *p* = 0.045; haemoglobin: 14.7 ± 0.96 vs. 13.8 ± 1.07 g/dL; effect size = −0.906; *p* < 0.001), whereas ferritin showed the opposite pattern, being higher in patients with a shorter duration (351 [193–493] vs. 219 [131–295] ng/mL; effect size = 0.920; *p* < 0.001). Electrical activity of the RVL and LVL was also higher in men with a shorter duration of long COVID (RVL: 27.0 ± 5.7 vs. 20.7 ± 4.5; effect size = 1.194; *p* = 0.036; LVL: 28.0 ± 5.6 vs. 22.7 ± 2.2; effect size = 1.128; *p* = 0.046). Regarding hospitalisation status, men hospitalised during the acute phase exhibited higher ferritin levels (331 [198–471] vs. 223 [130–310] ng/mL; effect size = 0.406; *p* = 0.009) and lower levels of haemoglobin (14.0 ± 1.11 vs. 14.6 ± 1.03 g/dL; effect size = 0.556; *p* = 0.034) and muscle mass (32.3 ± 2.8% vs. 34.1 ± 2.9%; effect size = 0.603; *p* = 0.017) compared to non-hospitalised men.

Taken together, these exploratory findings suggest that the pattern of between-group differences in laboratory and neuromuscular outcomes, according to long COVID duration and hospitalisation status, was more frequently observed among men than among women in this sample.

Table 3 summarises the PERMANOVA results for the 35 participants with complete data for the six-variable neuromuscular outcome matrix, comprising muscle mass percentage (MM), handgrip strength (HGS), and normalised RMS values for the right and left anterior deltoid (AD) and vastus lateralis (VL) muscles. No values in the neuromuscular response matrix were imputed. Analyses were repeated across five multiply imputed datasets for the laboratory biomarkers. Five of the eight biomarkers assessed—ferritin, calcium, potassium, haemoglobin, and LDH—showed significant marginal associations with the multivariate neuromuscular outcome in all five imputed datasets. Ferritin showed the largest median magnitude of association (partial R^2^ = 0.042; pseudo-*F* = 7.80), whereas the remaining significant biomarkers showed smaller and broadly similar associations (partial R^2^ range: 0.018–0.032). Despite their consistency across imputations, these effect sizes were small, with each biomarker individually explaining a limited proportion of the multivariate variance in neuromuscular outcomes. Given the restricted sample size, these findings should be interpreted as exploratory.

**Figure 2:**
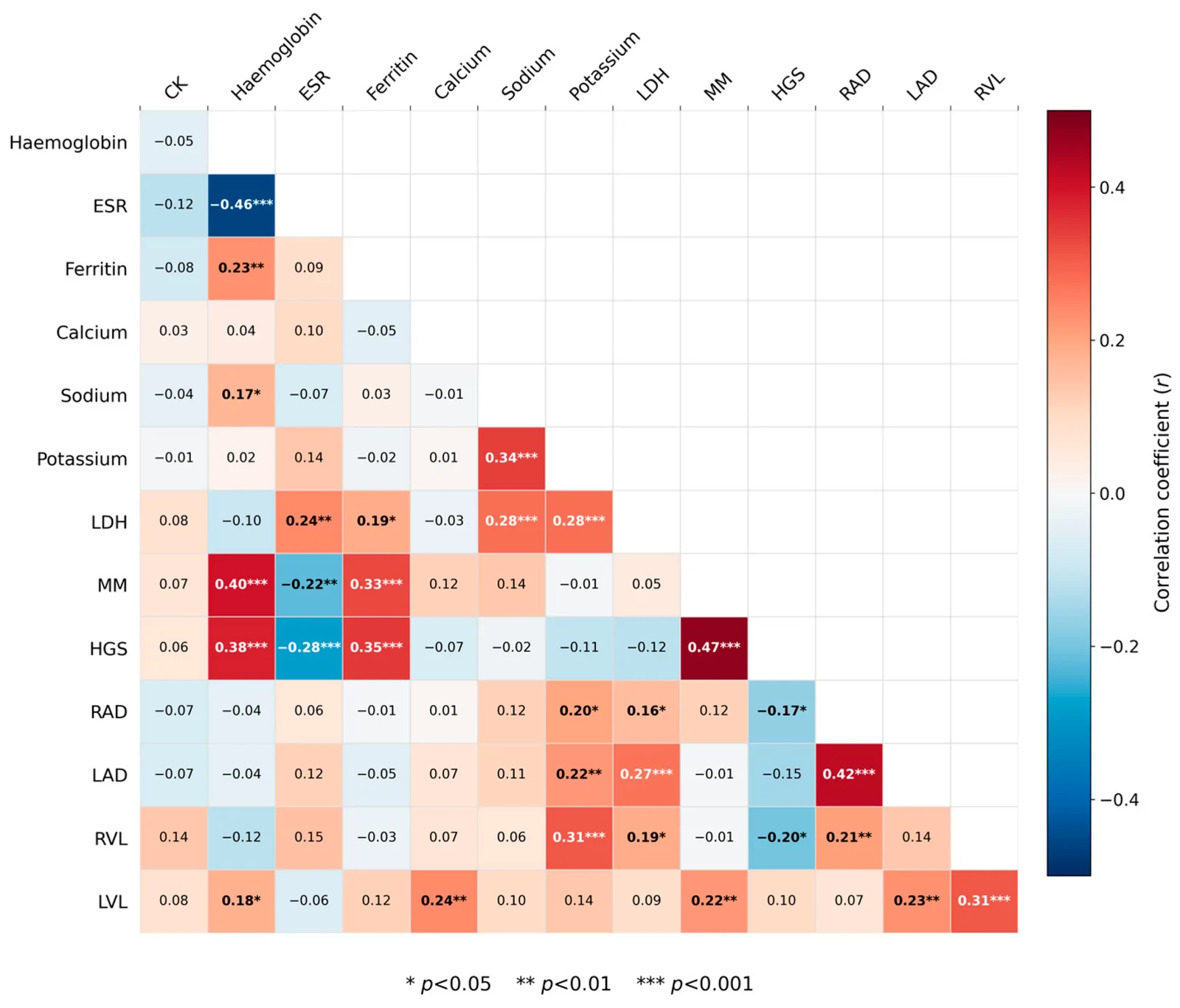
Correlation matrix between laboratory biomarkers and neuromuscular outcome measures. Statistical analysis: Pearson’s or Spearman’s correlation tests were applied based on the data’s normality. Cell colour and annotated values represent the correlation coefficient (r). The colour scale is centred on zero: white indicates no correlation (r = 0), whereas red and blue shades indicate positive and negative correlations, respectively, with colour intensity proportional to the strength of the correlation. Statistically significant correlations are shown in bold. Blank cells in the upper triangle were omitted, as they would duplicate the values in the lower triangle. CK: creatine kinase; ESR: erythrocyte sedimentation rate; LDH: lactate dehydrogenase; MM: percentage of muscle mass; HGS: hand grip strength; RAD: right anterior deltoid; LAD: left anterior deltoid; RVL: right vastus lateralis; LVL: left vastus lateralis. (*): *p* < 0.05; (**): *p* < 0.01; (***): *p* < 0.001.

Figure 2 presents the correlation heatmap between laboratory biomarkers and neuromuscular outcome variables. The strongest correlations observed were among the neuromuscular variables themselves—muscle mass and handgrip strength (r = 0.47, *p* < 0.001) and right and left anterior deltoid RMS (r = 0.42, *p* < 0.001), both of moderate magnitude, consistent with these variables representing related dimensions of muscle status.

Among laboratory biomarkers, haemoglobin, ferritin, and ESR showed the most consistent correlations with muscle mass and handgrip strength, of moderate magnitude. Haemoglobin and ferritin were positively correlated with muscle mass (r = 0.40 and 0.33, respectively; *p* < 0.001) and handgrip strength (r = 0.38 and 0.35, respectively; *p* < 0.001), while ESR was negatively correlated with HGS (r = −0.28, *p* < 0.001, weak magnitude) and, more weakly, with muscle mass (r = −0.22, *p* < 0.01). Haemoglobin and ESR were also correlated with one another, of moderate magnitude (r = −0.46, *p* < 0.001).

Electrolytes and LDH showed weak correlations, observed more consistently with muscle electrical activity (RMS) than with muscle mass or handgrip strength. Potassium was correlated with sodium (r = 0.34, *p* < 0.001, moderate magnitude) and, more weakly, with RMS of the anterior deltoid and vastus lateralis muscles (r ranging from 0.20 to 0.31, *p* < 0.05). LDH was weakly correlated with sodium, potassium, and RMS of the anterior deltoid muscles (r ranging from 0.16 to 0.28, *p* < 0.05).

Overall, the correlations observed across the matrix ranged from weak to moderate. Nonetheless, the consistency of the haemoglobin–ferritin–ESR axis with muscle mass and handgrip strength, together with the corresponding findings from the PERMANOVA analysis, reinforces the relevance of this pattern within the exploratory scope of the study. Given the cross-sectional design, these correlations describe the pattern of co-variation among variables at a single time point and should be interpreted as hypothesis-generating.

## 4. Discussion

This study investigated, in an exploratory and hypothesis-generating manner, the associations between laboratory biomarkers and neuromuscular outcomes in patients with long COVID, stratified by sex, long COVID duration, and hospitalisation status. Overall, between-group differences in laboratory and neuromuscular variables were observed more frequently among men than among women. Furthermore, a subset of biomarkers (ferritin, calcium, potassium, haemoglobin, and LDH) showed a consistent, albeit modest, association with the multivariate profile of neuromuscular outcomes across the multiply imputed datasets. In the correlation analysis, the haemoglobin–ferritin–ESR axis exhibited the most consistent correlations with muscle mass and handgrip strength, ranging from weak to moderate in magnitude, a pattern that was also reflected in the multivariate analysis.

In this sample, men more frequently required hospitalisation, intensive care unit admission, and invasive mechanical ventilation during the acute phase of COVID-19, and had longer hospital stays than women, whereas women reported a higher frequency of several long COVID symptoms (dyspnoea, myalgia, headache, chest pain, and lower limb oedema), as well as a longer mean duration, in months, of long COVID, compared with men, despite a less severe acute presentation. A similar divergence between acute-phase severity and long COVID symptom burden by sex has been described elsewhere in the literature [19], attributed to immunological and hormonal differences between sexes [20].

However, this pattern does not directly align with the between-subgroup differences observed in Table 2: when comparing the long COVID duration and hospitalisation subgroups separately in men and women, a greater number of laboratory and neuromuscular variables showed significant differences among the male subgroups than among the female subgroups. This apparent discrepancy suggests that acute-phase severity indicators alone are unlikely to fully account for the sex-specific pattern identified in this study. The imbalance in hospitalisation proportions between long COVID duration subgroups, present in both sexes, is a factor that warrants consideration as a possible additional contributor to the pattern observed.

Among the laboratory biomarkers assessed, the haemoglobin–ferritin–ESR axis showed the most consistent association with neuromuscular outcomes, both in the bivariate correlation analysis and in the multivariate PERMANOVA analysis. Lower haemoglobin and ferritin, and higher ESR, corresponded to lower muscle mass and lower handgrip strength in this sample, with a weak to moderate magnitude of association. Persistent disturbances in ferritin regulation have been repeatedly implicated in long COVID, particularly in relation to fatigue, exercise intolerance, and metabolic dysfunction [7]. Similarly, elevated ESR, together with altered ferritin, reduced haemoglobin, and elevated LDH, has been reported in patients with persistent fatigue and muscle weakness following COVID-19, a pattern described as compatible with an ongoing inflammatory/hypercatabolic state [21]. Previous studies have also reported an association between haemoglobin and oxygen transport capacity, suggesting that reduced oxygen availability may impair muscle oxidative metabolism and contribute to fatigue during submaximal exertion, as observed in studies using cardiopulmonary exercise testing [22] and near-infrared spectroscopy in individuals with long COVID [23,24]. The findings of the present study are compatible with this body of evidence, although the cross-sectional design does not allow testing of whether the associations identified here reflect an ongoing inflammatory process, a pre-existing condition related to iron stores and oxygen transport capacity, or other unassessed factors. The convergence between the two statistical methods employed, bivariate and multivariate, reinforces the consistency of the pattern observed in this sample, although its weak-to-moderate magnitude and the exploratory nature of the analyses indicate that this pattern should be interpreted as a preliminary finding, relevant for guiding future investigations into the relationship between these biomarkers and neuromuscular outcomes in long COVID.

A second, more discrete pattern related potassium and LDH to the muscle electrical activity of the AD and VL muscles, with weak-to-moderate magnitude in both the correlation analysis and the PERMANOVA. This pattern was concentrated predominantly in muscle electrical activity, differing from that observed for the haemoglobin–ferritin–ESR axis, which suggests that different biomarkers may relate to different dimensions of the neuromuscular outcome assessed in this sample. Given the weak magnitude of these associations and the absence of specific literature relating potassium and LDH to muscle electrical activity in patients with long COVID, this finding should be interpreted as preliminary, representing a still underexplored avenue that future studies may help clarify.

This study has several limitations. The cross-sectional design precludes inferences about direction, temporality, or causality, and the large number of comparisons performed without formal correction gives the analyses an exploratory character; their results should be interpreted as hypothesis-generating. The proportion of hospitalisation was not balanced between the long COVID duration subgroups in either sex, which may have contributed to part of the differences observed between these subgroups, independently of duration itself.

The absence of a healthy control group without long COVID limits the interpretation of our findings. Therefore, we cannot determine whether the observed neuromuscular patterns are specific to long COVID or may reflect sedentary behaviour, physical deconditioning, or other unmeasured factors. In this context, all participants were sedentary, which may have influenced body composition, muscle strength, and electromyographic findings independently of long COVID. Nevertheless, this characteristic may reflect the clinical profile of many individuals with long COVID, in whom persistent fatigue and exercise intolerance frequently contribute to physical inactivity.

Patients with pre-existing musculoskeletal conditions were excluded from this study to reduce diagnostic overlap with the neuromuscular outcomes assessed. This exclusion, although methodologically necessary, renders the sample more homogeneous and may not represent patients with long COVID who also present with musculoskeletal comorbidities. In the electromyographic assessment specifically, participants with overweight and obesity were excluded owing to the interference of subcutaneous adipose tissue with the surface EMG signal, resulting in a reduced subsample (*n* = 35 of 153); the EMG findings are therefore limited to individuals with normal body composition.

The multivariate analysis was restricted to the 35 participants with complete EMG data. This reduced sample size may have limited statistical power and the precision and stability of the PERMANOVA estimates. In addition, because participants with available EMG data may differ from those without such data, selection bias cannot be excluded. Consequently, the multivariate findings should be regarded as exploratory.

Taken together, the findings of this exploratory study suggest a sex-specific pattern in the laboratory and neuromuscular outcomes of long COVID, more frequently observed among men, with the haemoglobin–ferritin–ESR axis showing the most consistent association with muscle mass and handgrip strength. Given the cross-sectional and exploratory design, these results should be interpreted as hypothesis-generating rather than as causal evidence. Longitudinal studies with larger, more balanced samples could help clarify whether this axis precedes, accompanies, or follows the neuromuscular changes observed in long COVID, and further explore the sex-specific pattern and potential confounding factors identified here. By bringing together a multidimensional assessment that remains underexplored in the long COVID literature, this study offers a relevant basis for such future research.

## Figures and Tables

**Table 1:** Demographic and clinical characteristics of Long COVID Patients Stratified by Sex.

Variables (*n*, %)	Female (*n* = 83)	Male (*n* = 70)	*p* Value	Total (*n* = 153)
Demographic characteristics				
Age (years), mean ± SD	46.9 ± 11.9	49.8 ± 13.3	0.274	48.2 ± 12.6
Monthly income (BRL), *n*, (%)				
≤1 minimum wage	24 (28.9)	6 (8.6)	0.002 *	30 (19.7)
>1 minimum wage	59 (71.1)	54 (91.4)		123 (80.3)
Schooling, *n*, (%)				
Up to 9 years of study	5 (6.0)	14 (20.0)	0.009 *	19 (12.4)
More than 9 years of study	78 (94.0)	56 (80.0)		134 (87.6)
Lifestyle, *n*, (%)				
Current smoker	1 (1.2)	1 (1.4)	0.903	2 (1.3)
Former smoker	14 (16.9)	22 (31.4)	0.034 *	36 (23.5)
Clinical characteristics				
Body mass index, mean ± SD	29.8 ± 7.2	28.2 ± 4.3	0.117	29.1 ± 6.1
Hypertension, *n* (%)	16 (19.3)	24 (34.3)	0.035 *	40 (26.1)
Diabetes mellitus, *n* (%)	12 (14.5)	12 (17.1)	0.649	24 (15.7)
Obesity, *n* (%)	34 (41.0)	22 (31.4)	0.223	56 (36.6)
Hospitalisation during acute COVID-19				
Hospitalisation, *n* (%)	22 (26.5)	40 (57.1)	<0.001 *	62 (40.5)
Length of hospital stay (days), mean, SD	3.69 ± 8.9	13.7 ± 19.5	<0.001 *	8.29 ± 15.5
Intensive care unit admission, *n* (%)	0	13 (18.6)	<0.001 *	13 (8.5)
Invasive mechanical ventilation, *n* (%)	0	8 (11.4)	0.002 *	8 (5.2)
COVID-19 severity				
Non-severe	61 (73.5)	30 (42.9)	<0.001 *	91 (59.5)
Severe	22 (26.5)	40 (57.1)		62 (40.5)
Long COVID characteristics				
Duration (months), mean ± SD	8.51 ± 5.1	6.03 ± 5.3	0.004 *	7.37 ± 5.4
Self-reported symptoms, *n* (%)				
Fatigue	74 (89.2)	56 (80.0)	0.114	130 (85.0)
Dyspnoea	75 (90.4)	55 (78.6)	0.042 *	130 (85.0)
Muscle weakness	66 (79.5)	49 (70.0)	0.175	115 (75.2)
Myalgia	61 (73.5)	39 (55.7)	0.021 *	100 (65.4)
Headache	59 (71.1)	30 (42.9)	<0.001 *	89 (58.2)
Balance alterations	48 (57.8)	36 (51.4)	0.428	84 (54.9)
Chest pain	58 (69.9)	29 (41.4)	<0.001 *	87 (56.9)
Insomnia	43 (51.8)	32 (45.7)	0.453	75 (49.0)
Visual alterations	37 (44.6)	33 (47.1)	0.751	70 (45.8)
Tremble	31 (37.3)	28 (40.0)	0.737	59 (38.6)
Cough	34 (41.0)	21 (30.0)	0.159	55 (35.9)
Lower limb edema	34 (41.0)	14 (20.0)	0.005 *	48 (31.4)
Muscle symptom burden score				
0–1 symptoms, *n* (%)	5 (6.0)	14 (20.0)	0.009 *	19 (12.4)
≥2 symptoms, *n* (%)	78 (94.0)	56 (80.0)		134 (87.6)

Statistical analysis: Continuous variables were tested for normality using Shapiro–Wilk and compared using Student’s *t*-test or Mann–Whitney as appropriate. Categorical variables were compared using Fisher’s Exact Test or chi-square test. Significance level (*): *p* < 0.05. Muscle symptom burden score: burden of self-reported musculoskeletal symptoms. During 2020–2021, the Brazilian minimum wage ranged from BRL 1045 to BRL 1100, corresponding to approximately USD 203–212.

**Table 2:** Comparison of muscle and laboratory variables by sex, long COVID duration, and hospitalisation.

**Variables**	**Female ^a^**			
	**Comparison by Long COVID Duration**		**Comparison by Hospitalisation Status**	
	**Effect Size**	***p* Value**	**Effect Size**	***p* Value**
Laboratory analysis				
CK (U/L)	0.082	0.572	−0.114	0.457
LDH (U/L)	−0.106	0.466	−0.109	0.477
Ferritin (ng/mL)	0.041	0.784	0.227	0.144
Haemoglobin (g/dL)	−0.408	0.098	0.044	0.772
ESR (mm/h)	−0.286	0.044 *	−0.218	0.141
Calcium (mg/dL)	−0.128	0.469	0.017	0.930
Sodium (mEq/L)	−0.049	0.753	0.151	0.588
Potassium (mEq/L)	0.019	0.906	0.144	0.598
Clinical analysis				
Muscle mass (%)	−0.065	0.630	−0.393	0.008 *
Body fat mass (%)	−0.025	0.856	0.380	0.010 *
HGS (Kgf)	0.262	0.267	0.339	0.188
RMS (%) ^c^				
RAD	<0.001	0.999	−0.288	0.646
LAD	−0.451	0.315	0.546	0.388
RVM	0.141	0.750	−0.024	0.969
LVM	0.021	0.963	−0.318	0.613
RVL	−0.276	0.536	1.013	0.117
LVL	0.157	0.723	−0.789	0.216
**Variables**	**Male ^b^**			
	**Comparison by Long COVID Duration**		**Comparison by Hospitalisation Status**	
	**Effect Size**	***p* Value**	**Effect Size**	***p* Value**
Laboratory analysis				
CK (U/L)	0.299	0.045 *	−0.099	0.512
LDH (U/L)	−0.274	0.064	0.159	0.289
Ferritin (ng/mL)	0.920	<0.001 *	0.406	0.009 *
Haemoglobin (g/dL)	−0.906	<0.001 *	0.556	0.034 *
ESR (mm/h)	−0.252	0.087	0.255	0.088
Calcium (mg/dL)	−0.114	0.688	0.228	0.177
Sodium (mEq/L)	−0.378	0.184	0.139	0.622
Potassium (mEq/L)	0.030	0.861	0.095	0.560
Clinical analysis				
Muscle mass (%)	−0.077	0.757	0.603	0.017 *
Body fat mass (%)	−0.108	0.443	0.276	0.051
HGS (Kgf)	0.213	0.383	−0.104	0.461
RMS (%) ^c^				
RAD	0.196	0.710	0.318	0.547
LAD	0.859	0.119	−0.286	0.588
RVM	1.093	0.053	−0.010	0.984
LVM	0.901	0.103	−0.445	0.403
RVL	1.194	0.036 *	−0.062	0.960
LVL	1.128	0.046 *	0.210	0.691

Statistical analysis: Continuous variables were tested for normality using the Shapiro–Wilk test and compared using Student’s *t*-test or Mann–Whitney U test, as appropriate. Effect sizes are presented as Cohen’s d (for parametric variables) or rank-biserial correlation (for non-parametric variables). Significance level (*): *p* < 0.05. Comparison by long COVID duration: participants with long COVID lasting up to 6 months (G ≤ 6) were compared with those with long COVID lasting more than 6 months (G > 6). Comparison by hospitalisation status: participants who were hospitalised during the acute phase of COVID-19 (GH) were compared with those who were not hospitalised (GNH). CK: creatine kinase. LDH: lactic dehydrogenase. ESR: Erythrocyte Sedimentation Rate. U/L: units per liter. ng/mL: nanograms per milliliter. HGS: hand grip strength. KgF: kilogram force. RMS: root mean square. RAD: right anterior deltoid. LAD: left anterior deltoid. RVM: right vastus medialis. LVM: left vastus medialis. RVL: right vastus lateralis. LVL: left vastus lateralis. ^a^: Number of women who did not undergo examinations: G ≤ 6: CK/LDH (5), SF (6), bioimpedance (2), HGS (2); G > 6: CK (13), LDH (8), ferritin (9), bioimpedance (1), HGS (3); GNH: CK/LDH (14), SF (15), bioimpedance (4), dynamometry (6). ^b^: Number of men who did not undergo examinations: G ≤ 6: CK/LDH (6), SF (7), bioimpedance (2); G > 6: CK (2), LDH (1), ferritin (4), bioimpedance (2); GH: CK (4), LDH (3), SF (15), bioimpedance (4), dynamometry (6); GNH: CK/LDH (4), SF (5), bioimpedance (3). ^c^: Patients included for kinesiological electromyography: women—G ≤ 6 (6), G > 6 (15); GH (5), GNH (16); men—G ≤ 6 (8), G > 6 (6); GH (5), GNH (9). Complete descriptive statistics for the analysed variables are presented in Supplementary Table S2.

**Table 3:** PERMANOVA results based on five multiple imputations.

Biomarker	Median Partial R^2^ (Range)	Median Pseudo-F (Range)	Imputed Datasets with *p* < 0.05, *n*/5	Median *p* Value (Range)
CK	0.005 (0.005–0.012)	0.88 (0.85–2.29)	0/5	0.050–0.497
Calcium	0.032 (0.017–0.035)	6.04 (3.22–6.63)	5/5	<0.001–0.013
ESR	0.009 (0.007–0.013)	1.75 (1.35–2.43)	2/5	0.034–0.236
Ferritin	0.042 (0.037–0.046)	7.80 (6.69–8.74)	5/5	<0.001–<0.001
Hemoglobin	0.022 (0.018–0.027)	4.11 (3.38–5.07)	5/5	<0.001–0.007
LDH	0.018 (0.015–0.024)	3.36 (2.67–4.50)	5/5	0.002–0.028
Potassium	0.023 (0.019–0.030)	4.19 (3.61–5.56)	5/5	<0.001–0.006
Sodium	0.005 (0.002–0.011)	0.84 (0.34–2.14)	0/5	0.063–0.891

Notes: The PERMANOVA analysis was restricted to participants with complete EMG data (*n* = 35). The multivariate response matrix included muscle mass percentage, handgrip strength, and normalized root mean square values for the right and left anterior deltoid and vastus lateralis muscles. All biomarkers were entered simultaneously into a single PERMANOVA model and tested marginally using adonis2 (by = “margin”). Analyses were repeated across five multiply imputed datasets. Reported medians and ranges describe stability across imputations; they are not formally pooled permutation-based estimates. CK: Creatine kinase. LDH: Lactate dehydrogenase. ESR: Erythrocyte Sedimentation Rate. Results for each of the five imputed datasets are presented in Supplementary Table S3.

## Data Availability

The raw data supporting the conclusions of this article will be made available by the authors on request.

## Supplementary Materials

The following supporting information can be downloaded at: https://www.mdpi.com/article/10.3390/v18101099/s1, Table S1: Reference values for bioelectrical impedance analysis; Table S2: Comparison of muscle and laboratory variables by sex, long COVID duration, and hospitalisation; Table S3: PERMANOVA results for each of the five imputed datasets.
