# Supplementary material for: Neuromuscular Phenotype in Long COVID Is Associated with Inflammation, Mineral Homeostasis, and Muscle Metabolism
Source: Viruses. 2026 Oct 4;18(10):1099. doi: 10.3390/v18101099 (PMC13709923; doi:10.3390/v18101099)
Supplement: Supplementary file 1 [file viruses-18-01099-s001.zip › Supplementary Table S1.pdf]

Supplementary table S1. Reference values for bioelectrical impedance analysis.

**Interpreting the Body Mass Index (BMI) Result**	
BMI	*BMI classification**
Less than 18.5	Underweight
18.5 or more and less than 25	Normal
25 or more and less than 30	Overweight
30 or more	Obese
**Interpreting the Body Fat Percentage Result**	
Female Sex /Age	*Normal*
20-39	21.0 - 32.9
40-59	23.0 - 33.9
60-79	24.0 - 35.9
Male Sex /Age	*Normal*
20-39	8.0 - 19.9
40-59	11.0 - 21.9
60-79	13.0 - 24.9
**Interpreting the Skeletal Muscle Percentage Result**	
Female Sex /Age	*Normal*
18-39	24.3 - 30.3
40-59	24.1 - 30.1
60-80	23.9 - 29.9
Male Sex /Age	*Normal*
18-39	33.3 - 39.3
40-59	33.1 - 39.1
60-80	32.9 - 38.9

*: BMI classification according to the World Health Organization (WHO). Reference values recommended by the manufacturer for the bioelectrical impedance analysis. Adapted from the Omron® instruction manual for the Full Body Sensor Body Composition Monitor and Scale, Model HBF-514C.
