# Supplementary material for: Neuromuscular Phenotype in Long COVID Is Associated with Inflammation, Mineral Homeostasis, and Muscle Metabolism
Source: Viruses. 2026 Oct 4;18(10):1099. doi: 10.3390/v18101099 (PMC13709923; doi:10.3390/v18101099)
Supplement: Supplementary file 1 [file viruses-18-01099-s001.zip › Supplementary Table S2.pdf]

Supplementary Table S2. Comparison of muscle and laboratory variables by sex, long COVID duration, and hospitalisation.

Female[a]								
**Variables**	**G≤6 (n = 20)**	**G> 6 (n = 63)**	*Efect size*	*P* value	**Hospitalised (n = 22)**	**Non-hospitalised (n = 61)**	*Efect size*	*P value*
**Laboratory analysis, Median (IQR)**								
CK (U/L)	75.5 [57 – 84]	79.5 [58 – 118]	0.082	0.572	73.0 [49 – 88]	79.0 [58 – 113]	-0.114	0.457
LDH (U/L)	322.0 [275 – 288]	322.0 [265 – 370]	-0.106	0.466	310.0 [267 – 353]	328.0 [270 – 379]	-0.109	0.477
Ferritin (ng/Ml)	85.0 [40 – 155]	106.0 [59 – 148]	0.041	0.784	128.0 [78 – 161]	87.5 [46 – 147]	0.227	0.144
Haemoglobin (g/dL)	12.4±1.28	12.9±0.96	-0.408	0.098	13.0 [11.7–13.8]	12.9 [12.2–13.3]	0.044	0.772
ERS (mm/h)	55.5 [30.8–66.8]	42.0 [25.8–49.3]	-0.286	0.044*	34.5 [26.5–51.0]]	42.5 [32.0 –61.3]	-0.218	0.141
Calcium (mg/dL)	9.90 [9.5 – 10.4]	9.75 [9.4 – 10.2]	-0.128	0.469	9.80 [9.40 – 10.3]	9.80 [9.45 – 10.2]	0.017	0.930
Sodium (mEq/L)	142 [139 – 143]	141 [139 – 143]	-0.049	0.753	141 [139 – 142]	142 [139 – 143]	0.151	0.588
Potassium (mEq/L)	4.05 [3.8 – 4.3]	4.10 [3.9 – 4.3]	0.019	0.906	4.0 [3.9 – 4.3]	4.10 [3.9 – 4.4]	0.144	0.598
**Clinical analysis, Median (IQR)**								
Muscle mass (%)	24.0 [22.2 – 25.4]	23.0 [21.8 – 24.9]	-0.065	0.630	22.3 [21.1 – 23.5]	23.9 [22.4 – 25.6]	-0.393	0.008*
Body fat mass (%)	41.5 [37.6 – 49.2]	42.8 [37.0 – 49.0]	-0.025	0.856	48.0 [44.0 – 50.7]	40.5 [35.8 – 47.8]	0.380	0.010*
HGS (Kgf)	12.0 [9 – 16]	11.0 [8 – 14]	0.262	0.267	11 [8 – 14]	12 [9 – 15]	0.339	0.188
RMS (%)[c]								
RAD	34.6±7.6	34.6±6.6	<.001	0.999	36.4±8.8	34.4±6.7	-0.288	0.646
LAD	35.7±5.1	38.4±6.5	-0.451	0.315	34.6±7.3	37.9±5.9	0.546	0.388
RVM	24.3±3.2	23.6±5.8	0.141	0.750	24.0±7.3	23.9±4.8	-0.024	0.969
LVM	21.9±9.1	21.8±6.9	0.021	0.963	23.9±9.8	21.5±7.5	-0.318	0.613
RVL	24.2±4.9	26.0±7.2	-0.276	0.536	20.0±5.8	26.2±6.2	1.013	0.117
LVL	23.7±7.2	22.7±5.6	0.157	0.723	27.2±12.1	22.5±4.9	-0.789	0.216
**Male[b]**								
**Variables**	**G≤6 (n = 36)**	**G> 6 (n = 34)**	*Efect size*	*P* value	**GH (n = 40)**	**GNH (n = 30)**	*Efect Size*	*P value*
**Laboratory analysis**								
CK (U/L)	69 [51.5 – 124]	100 [79 – 146]	0.299	0.045*	83.0 [59.5 – 116]	98.5 [60 – 140]	-0.099	0.512
LDH (U/L)	335 [283 – 398]	301 [266 – 342]	-0.274	0.064	328 [276 – 371]	306 [268 – 347]	0.159	0.289
Ferritin (ng/Ml)	351 [193 – 493]	219 [131 – 295]	0.920	<0.001*	331 [198 – 471]	223 [130 – 310]	0.406	0.009*
Haemoglobin (g/dL)	13.8±1.07	14.7±0.96	-0.906	<0.001*	14.0±1.11	14.6±1.03	0.556	0.034*
ERS (mm/h)	32 [15.8–49.8]	20 [12.0–33.0]	-0.252	0.087	30.0 [18.0 –52.0]	18.5 [12.0–41.0]	0.255	0.088
Calcium (mg/dL)	9.9 [9.7 – 10.4]	10.0 [9.7 – 10.5]	-0.114	0.688	10.0 [9.7 – 10.6]	9.8 [9.6 – 10.1]	0.228	0.177
Sodium (mEq/L)	142 [139 – 144]	142 [141 – 145]	-0.378	0.184	142± 4.1	142± 2.7	0.139	0.622
Potassium (mEq/L)	4.0 [3.9 – 4.2]	4.05 [3.9 – 4.3]	0.030	0.861	4.05 [3.9 – 4.3]	4.0 [3.9 – 4.2]	0.095	0.560
**Clinic analysis**								
Muscle mass (%)	32.7 [30.6 – 34.5]	33.3 [31.6 – 34.5]	-0.077	0.757	32.3±2.8	34.1±2.9	0.603	0.017*

Body fat mass (%)	28.5 [23.8 – 32.6]	27.8 [23.3 – 31.3]	-0.108	0.443	29.6 [24.6 – 33.2]	26.8 [22.9 – 30.0]	0.276	0.051
HGS (Kgf)	23.0 [20 -28]	22.0 [19 – 25.8]	0.213	0.383	22 [20 – 25]	24 [19 – 28]	-0.104	0.461
RMS (%)[c]								
RAD	35.0±7.8	33.7±4.2	0.196	0.710	35.3±5.2	33.2±8.8	0.318	0.547
LAD	37.2±5.7	32.9±3.6	0.859	0.119	35.0±6.5	36.6±2.9	-0.286	0.588
RVM	25.5±4.9	20.2±4.7	1.093	0.053	23.5±5.7	23.6±5.4	-0.010	0.984
LVM	25.8±4.9	21.9±3.2	0.901	0.103	23.6±3.2	25.7±6.6	-0.445	0.403
RVL	27.0±5.7	20.7±4.5	1.194	0.036*	24.6±5.9	24.7±6.7	-0.062	0.960
LVL	28.0±5.6	22.7±2.2	1.128	0.046*	26.4±5.8	25.3±4.4	0.210	0.691

Statistical analysis: Continuous variables were tested for normality using the Shapiro-Wilk test and compared using Student's t-test or Mann-Whitney U test, as appropriate. Effect sizes are presented as Cohen's d (for parametric variables) or rank-biserial correlation (for non-parametric variables). Significance level (*): $p < 0.05$. G ≤ 6: patients with long COVID lasting up to 6 months; G > 6: patients with long COVID lasting more than 6 months. GH: hospitalized patients' group. GNH: non-hospitalized patients' group.CK: creatine kinase. LDH: lactic dehydrogenase. ERS: U/L: units per liter. ng/Ml: nanograms per milliliter. HGS: hand grip strength. KgF: kilogram force. RMS: root mean square. RAD: right anterior deltoid. LAD: left anterior deltoid. RVM: right vastus medialis. LVM: left vastus medialis. RVL: right vastus lateralis. LVL: left vastus lateralis. a: Number of women who did not undergo examinations: G ≤ 6: CK/LDH (5), SF (6), bioimpedance (2), HGS (2); G > 6: CK (13), LDH (8), ferritin (9), bioimpedance (1), HGS (3); GNH: CK/LDH (14), SF (15), bioimpedance (4), dynamometry (6). b: Number of men who did not undergo examinations: G ≤ 6: CK/LDH (6), SF (7), bioimpedance (2); G > 6: CK (2), LDH (1), ferritin (4), bioimpedance (2); GH: CK (4), LDH (3), SF (15), bioimpedance (4), dynamometry (6); GNH: CK/LDH (4), SF (5), bioimpedance (3). c: Patients included for kinesiological electromyography: women – G ≤ 6 (6), G > 6 (15); GH (5), GNH (16); men – G ≤ 6 (8), G > 6 (6); GH (5), GNH (9).
