# Supplementary material for: Neuromuscular Phenotype in Long COVID Is Associated with Inflammation, Mineral Homeostasis, and Muscle Metabolism
Source: Viruses. 2026 Oct 4;18(10):1099. doi: 10.3390/v18101099 (PMC13709923; doi:10.3390/v18101099)
Supplement: Supplementary file 1 [file viruses-18-01099-s001.zip › Supplementary Table S3.pdf]

Supplementary Table S3. PERMANOVA results for each of the five imputed datasets.

Imputed dataset	Biomarker	Degrees of freedom	Sum of squares	Partial R^2	Pseudo-F	Permutation p-value
Imputation 1	Calcium	1	31.7452	0.0348	6.631	<0.001
Imputation 1	CK	1	10.9738	0.0120	2.292	0.050
Imputation 1	ESR	1	11.5063	0.0126	2.403	0.043
Imputation 1	Ferritin	1	41.8510	0.0459	8.741	<0.001
Imputation 1	Haemoglobin	1	21.3468	0.0234	4.459	0.001
Imputation 1	LDH	1	21.5452	0.0236	4.500	0.002
Imputation 1	Potassium	1	17.2620	0.0189	3.606	0.006
Imputation 1	Sodium	1	2.9507	0.0032	0.616	0.671
Imputation 2	Calcium	1	29.6344	0.0325	6.041	<0.001
Imputation 2	CK	1	4.3347	0.0048	0.884	0.485
Imputation 2	ESR	1	7.5579	0.0083	1.541	0.166
Imputation 2	Ferritin	1	38.2438	0.0419	7.796	<0.001
Imputation 2	Haemoglobin	1	16.5753	0.0182	3.379	0.007
Imputation 2	LDH	1	15.0613	0.0165	3.070	0.015
Imputation 2	Potassium	1	27.2935	0.0299	5.564	<0.001
Imputation 2	Sodium	1	1.6502	0.0018	0.336	0.891
Imputation 3	Calcium	1	31.6239	0.0347	6.459	<0.001
Imputation 3	CK	1	6.1731	0.0068	1.261	0.283
Imputation 3	ESR	1	11.8950	0.0130	2.429	0.034
Imputation 3	Ferritin	1	40.3928	0.0443	8.249	<0.001
Imputation 3	Haemoglobin	1	18.1624	0.0199	3.709	0.004
Imputation 3	LDH	1	19.9926	0.0219	4.083	0.002

Imputed dataset	Biomarker	Degrees of freedom	Sum of squares	Partial R²	Pseudo-F	Permutation p-value
Imputation 3	Potassium	1	23.1116	0.0253	4.720	<0.001
Imputation 3	Sodium	1	4.1229	0.0045	0.842	0.498
Imputation 4	Calcium	1	28.1498	0.0309	5.656	<0.001
Imputation 4	CK	1	4.2186	0.0046	0.848	0.497
Imputation 4	ESR	1	6.7069	0.0074	1.348	0.236
Imputation 4	Ferritin	1	33.3101	0.0365	6.693	<0.001
Imputation 4	Haemoglobin	1	20.4434	0.0224	4.107	0.003
Imputation 4	LDH	1	13.3044	0.0146	2.673	0.028
Imputation 4	Potassium	1	20.8780	0.0229	4.195	0.002
Imputation 4	Sodium	1	4.2309	0.0046	0.850	0.499
Imputation 5	Calcium	1	15.7360	0.0173	3.217	0.013
Imputation 5	CK	1	4.1407	0.0045	0.847	0.490
Imputation 5	ESR	1	8.5826	0.0094	1.755	0.124
Imputation 5	Ferritin	1	37.7862	0.0414	7.725	<0.001
Imputation 5	Haemoglobin	1	24.7943	0.0272	5.069	<0.001
Imputation 5	LDH	1	16.4424	0.0180	3.362	0.007
Imputation 5	Potassium	1	18.3140	0.0201	3.744	0.006
Imputation 5	Sodium	1	10.4620	0.0115	2.139	0.063

Notes: Results are presented separately for each of the five datasets generated through multiple imputation. The multivariate response matrix comprised muscle mass percentage, handgrip strength, and normalised RMS values for the right and left anterior deltoid and right and left vastus lateralis muscles. All biomarkers were entered simultaneously into a single PERMANOVA model and tested marginally using adonis2(..., by = "margin"), with Euclidean distances calculated after standardisation of the response variables. *P*-values were obtained using 999 permutations. CK: creatine kinase; ESR: erythrocyte sedimentation rate; LDH: lactate dehydrogenase; RMS: root mean square.
